# Antibody response to melanoma helper-peptide vaccine correlates with survival

**DOI:** 10.1186/2051-1426-1-S1-P229

**Published:** 2013-11-07

**Authors:** Walter C Olson, Caroline M Reed, Craig L Slingluff

**Affiliations:** 1 Surgery, University of Virginia, Charlottesville, VA, USA

## Introduction/Background

A melanoma vaccine comprising 6 peptides presented by Class II MHC molecules was safe and induced CD4+ T cell responses in 81% of recipients. Circulating CD4+ T cell responses to these 6 melanoma helper peptides (6MHP) have been associated with patient survival. We hypothesized that these intermediate length peptides (14-23 amino acids) may also induce antibody responses, and that antibody responses may correlate with CD4+ T cell responses and with clinical outcome.

## Methods

The original trial enrolled 39 patients who were vaccinated with peptides in incomplete Freund’s adjuvant plus GM-CSF. Sera from 24 patients have been evaluated for antibodies to the 6MHP. Antibody titer was measured with an indirect enzyme-linked immunosorbent assay (ELISA) of serum taken before the first vaccine and after 4-6 vaccines (5-7 weeks after the first injection). A positive antibody response was defined as the reciprocal of the dilution that gives a fluorescent signal ten-fold greater than that of a control serum and is greater than 100. Analysis was performed using Kaplan-Meier curves, Chi-square analysis, and Spearman’s rank correlation with MedCalc software.

## Results

Antibody responses to the 6MHP with titers ranging from 100 to 10,889 (median titer = 2,157) were identified in 15 patients (58%), but were not detectable in 9 (42%). There was an association between antibody response and T cell response in the same blood samples (Spearman’s rho= 0.51; p = 0.0115); among patients with antibody response, 9/15 (60%) had a T cell response in the PBMC, but in those without antibody response, only 1/9 (11%) had a T cell response. Survival was strongly associated with antibody response, with median survival of 1.2 years in those without antibody response, but median survival not reached (greater than 5 years) for those with antibody response (p = 0.01).

## Discussion

Antibody responses to NY-ESO-1 are well-characterized, but the occurrence and significance of antibody responses to other melanoma antigens is not known. The peptides in this vaccine mixture are derived from gp100, MART-1/MelanA, tyrosinase, MAGE-A3 and other MAGE proteins. These intermediate length peptides, administered in incomplete Freund’s adjuvant plus GM-CSF, induce strong antibody responses. Concern that the antibodies might interfere with immunogenicity by binding the peptides and clearing them is not supported because the presence of antibody is associated with CD4+ T cell response. On the other hand, these antibody responses appear to be clinically significant because they are strongly associated with patient survival. It is conceivable that these antibodies could lead to antigen-antibody complexes that support antigen uptake and presentation by dendritic cells.

